# Lanthanum and abscisic acid coregulate chlorophyll production of seedling in switchgrass

**DOI:** 10.1371/journal.pone.0232750

**Published:** 2020-05-05

**Authors:** Xueqing He, Pei You, Yunfu Sun

**Affiliations:** College of Grassland Agriculture, Northwest A&F University, Yangling, Shaanxi Province, China; United Arab Emirates University, UNITED ARAB EMIRATES

## Abstract

The rare earth element lanthanum (La) has been proven to be beneficial for plant growth with a low concentration, and abscisic acid (ABA) which is a plant hormone also can regulate plant growth. In the present study, we investigated the germination and seedling growth of switchgrass (*Panicum virgatum* L.) under La (10 μM), ABA (10 μM) and La + ABA treatments. The results showed that La, ABA and La + ABA treatments could not significantly affect the germination and shoot length as compared to the control (*P*>0.05). However, La treatment increased the root activity and chlorophyll content, and ABA treatment enhanced root length and root activity (*P*<0.05). La + ABA treatments demonstrated that La could not significantly alleviate the promotion of ABA in root length, while ABA reversed the increase of chlorophyll content caused by La. The coregulation of La and ABA on chlorophyll content was further explored by in vitro experiments and quantum chemical calculations. In vitro experiments revealed that La, ABA, and La + ABA treatments reduced the absorbance of chlorophyll, and quantum chemical calculations indicated that the reduction of absorbance was caused by the reactions between La, ABA and chlorophyll. In vivo and in vitro experiments, together with quantum chemical calculations, demonstrated that both ABA and La could stimulate the production of chlorophyll, while they also could react with chlorophyll to produce La-monochlorophyll, La-bischlorophyll, and ABA adsorbed chlorophyll, which had lower absorbance. La + ABA treatment significantly decreased the chlorophyll content in vivo. This phenomenon was due to the fact that La and ABA formed LaABA compound, which markedly reduced the concentrations of ABA and La, and the effect of promoting chlorophyll production was overcome by the effect of reducing chlorophyll absorbance.

## Introduction

Rare earth elements (REEs) have been widely used as microfertilizers in agriculture [1–4] because they can improve plant growth and stress resistance at certain concentrations. Lanthanum (La) is a rare earth element that has been extensively studied due to its wide application in numerous fields and potential accumulation in the environment [5–7]. Previous studies have demonstrated that La induces hormesis in many plant physiological processes, such as seedling growth, peroxidase activity, net photosynthesis and chlorophyll production, which have a dose response characterized by stimulation at low concentrations and inhibition at high concentrations [8–11]. D’Aquino *et al*. [12] reported that La stimulated the root growth of *Triticum durum* at 0.01 and 0.1 mM after 9 d of treatment, however, it inhibited the root growth with a concentration equal to or larger than 1 mM. Shi *et al*. [13] showed that low concentrations (0.002–0.2 mM) of La increased the chlorophyll content in cucumber seedling leaves, while high concentration (2 mM) of La reduced the chlorophyll content. Oliveira *et al*. [14] found that the dry matter yield of shoot and root, photosynthetic rate and total chlorophyll content were promoted at low La concentrations (5 and 10 μM), whereas soybean growth was reduced at high La concentrations (80 and 160 μM). Although there is no exact concentration threshold for La to inhibit plant growth [6], it is apparent that La with a concentration of several μM can stimulate seedling growth.

Abscisic acid (ABA) is a plant hormone that regulates plant growth, development and reproduction, especially during environmental stresses [15–17]. ABA inhibits seed germination in most plant species and regulates the root growth [18]. Smet *et al*. [19] demonstrated that seed germination of *Arabidopsis* was inhibited completely by 3.0 μM ABA. Fujii *et al*. [20] found that root length of *Arabidopsis* was elongated by 0.5 μM ABA, while it was reduced to less than a half when ABA concentration was larger than 10 μM. Schnall *et al*. [21] showed that 10 μM ABA could reduce the number of long *Arabidopsis* root hairs by 45% as compared to control, and 500 μM ABA eliminated these long root hairs completely. Sarath *et al*. [22,23] concluded that ABA could potentially block nitric oxide-responsive cascades and inhibit germination but that hydrogen peroxide could overcome these effects. There is a complex relationship between La and ABA. Liu *et al*. [24] showed that the endogenous ABA in root of *Zea mays* had temporal changes when exposed the root to La. Wang *et al*. [25] found that 10 μM La could rescue the inhibited seed germination rate and root elongation growth in Arabidopsis caused by 1 μM exogenous ABA. So, ABA may play an import role in La regulation of plant growth. Multiple studies have proven that La affects plant growth via its synergistic and agonistic interactions with ABA [26–28].

Switchgrass (*Panicum virgatum* L.) is a perennial warm-season C_4_ grass that has been widely used as ground cover, as forage for livestock, for soil and water conservation, and for wildlife habitat restoration. In addition, established switchgrass stands are very resilient to environmental fluctuations [29]. Therefore, switchgrass is considered a resource-efficient, low-input crop for producing bioenergy on farmland [30]. Several studies have investigated the seed germination of switchgrass under La or ABA stress. Thomas *et al*. [31] found that La had no effect on germination of switchgrass at any dose, which was consistent with our previous work [32]. Duclos *et al*. [18] showed that ABA inhibited the germination of switchgrass at a dose equal to or larger than 10 μM. However, how La and ABA affect the seedling growth of switchgrass is not clear yet. In particular, whether La and ABA can regulate each other in seedling growth of switchgrass is unknown.

La may take part in the photosynthesis in switchgrass. Hong *et al*. [33] revealed that La could enter chloroplasts and coordinate with nitrogens of porphyrin ring to form La-chlorophyll in spinach leaves. Wang *et al*. [34] proposed that La could replace Mg and form [Chlorophyll-a · La · pheophytin-a] species in spinach leaves. These reactions in vivo are so complicated that it is hard to get the details. The quantum chemical methods provide an effective way to investigate these reactions at molecular level. With density functional theory (DFT) and time-dependent density functional theory (TD-DFT), Liao *et al*. [35] successfully predicted the molecular structures of lanthanide mono- and bisporphyrin complexes. Yin [36] calculated the free energies of deprotonated and protonated species of prophyrin, and showed which process was thermodynamically favorable. Barnsley *et al*. [37] reproduced the experimental UV-vis spectra of two aldehyde-porphyrin isomers by computing electronic absorption spectra. We would employ the quantum chemical calculations together with in vitro experiments to investigate the possible substitution reactions in chlorophyll.

Hence, in the present study, we investigated the effects of La and ABA on the seed germination and seedling growth of switchgrass by in vivo experiments. Some associated physiological mechanisms were also explored by in vitro experiments and quantum chemical calculations. Thus, the objectives of this study were to (1) investigate the possible use of La as a fertilizer during switchgrass growth and the specific mechanism of its interaction with ABA and (2) determine whether La and ABA coregulate the chlorophyll content of switchgrass. The results will help researchers in related fields better understand the biological effect of the rare earth element La on plant hormone (ABA) signal transduction in switchgrass, as well as provide new insights into agronomic management to reduce ABA accumulation in switchgrass products.

## Materials and methods

### Materials

Mature seeds of Alamo switchgrass were harvested in October 2017 from the Experimental Station of Grassland Science in Yangling (N34°16′, E 108°4′), Shaanxi Province, PR China. They were then cleaned and stored at 4°C in paper bags for later use. According to the standards for forage seed testing, the thousand-seed weight was 1.193 g, the seed viability was 98%, and the initial moisture content was 9.2%. ABA was purchased from Sigma company with a purity larger than 98.5%. La(NO_3_)_3_ · 6H_2_O was purchased from Aladdin company with a purity larger than 99.99%. Chlorophyll was purchased from Macklin company with a purity larger than 95%.

### Germination test

The plants were grown in a growth chamber at 25±2°C with 70%-85% humidity and a 16/8 h light/dark cycle with 75 μmol·m^2^/s irradiance. Seeds were surface sterilized using 75% alcohol for 30 s, sterilized using 5% NaClO for 2.5 h, rinsed with sterile water five times, and soaked in sterile water overnight. After 24 h, the seeds were resterilized using 5% NaClO for 30 min and rinsed with sterile water five times. To measure the seed germination, seeds were grown in tissue-culture vessels with 1/2 Murashige-Skoog (MS) medium whose concentration was reduced by half as compared to the original MS medium [38]. In our 1/2 MS medium, Edamin, Indoleacetic acid and Kinetin were removed, and the concentrations of agar and sucrose were set to 7 and 15 g/L, respectively. In addition, the pH was adjusted to 5.6 by KOH. A control (0 μM), La(NO_3_)_3_ (10 μM), ABA (10 μM), and La(NO_3_)_3_: ABA = 1:1 in stock solutions were added to the 1/2 MS medium at identical concentrations. They were three repliacions for each treatment (N = 50). The germination was defined as the emergence of the radical through the seed coat.

$$\text{Germination}\;\text{rate}(\%)=\frac{\text{Number}\;\text{of}\;\text{germinated}\;\;\text{seeds}\;\;\text{after}\;\;\text{n}\;\text{days}\;}{\text{Number}\;\text{of}\;\text{tested}\;\;\text{seeds}}\times 100\%\;\;\;\;(\text{n}=5,14)$$

### Seedling growth assay

The root length and shoot length were measured on 10 individuals per experiment after 14 d of seed germination. The root system activity was determined by using the triphenyl tetrazolium chloride (TTC) method as described in the literature [39]. Fresh leaves were soaked in 100% ethanol to extract chlorophyll and the chlorophyll content (Chl total) was determined by the method of Rowan [40,41] at wavelengths of 665 and 649 nm. The values of chlorophyll content have been expressed as mg/g of fresh weight (FW).

$$\text{Chl}\;\text{a}(\text{mg}/\text{L})=13.70\times {\text{A}}_{665}-5.76\times {\text{A}}_{649}$$

$$\text{Chl}\;\text{b}(\text{mg}/\text{L})=-7.60\times {\text{A}}_{665}+25.8\times {\text{A}}_{649}$$

$$\text{Chl}\;\text{total}(\text{mg}/\text{L})=6.10\times {\text{A}}_{665}+20.04\times {\text{A}}_{649}$$

Here, A_665_ and A_649_ are the absorbance of chlorophyll at 665 and 649 nm, respectively.

### In vitro experiments

In vitro experiments were performed, in which La(NO_3_)_3_ (10 μM), ABA (10 μM) or ABA: La(NO_3_)_3_ = 1:1 was added directly to the solution of Mg-chlorophyll compounds (Mg-chlorophyll A (MgCA) and Mg-chlorophyll B (MgCB)) with anhydrous ethanol as the solvent. UV-vis spectra of these solutions and the absorbance in the first 2 h were measured using a spectrophotometer (Shimadzu UV-3900 UV-VIS Spectrophotometer, Tokyo, Japan).

### Quantum chemical calculations

All the calculations were performed by applying the Gaussian 09 package (**Gaussian 09**) [42] with density functional theory (DFT). Geometric optimizations were performed using the M06 functional [43], where the Stuttgart-Dresden (SDD) basis set was applied for La and the 6-31G (d) basis set was employed for the other atoms. The solvent effect was taken into account by the polarizable continuum model (PCM) of the self-consistent reaction field (SCRF) procedure in ethanol. The Gibbs free energies were calculated for all of the optimized structures at 298.15 K and 101 kPa. Furthermore, the electronic absorption spectra were also calculated using the time-dependent density functional theory (TDDFT) with the optimized structures.

### Statistical analysis

A one-way analysis of variance (ANOVA) at a significance level *P*<0.05 was performed using SPSS 22.0 software. Duncan’s multiple range tests was used to compare means of germination potential, germination rate, root length, shoot length, root activity and total chlorophyll content between treatments when significant differences were found.

## Results

### Effects of La and ABA treatments on seed germination and seedling growth in switchgrass

**Fig 1:**
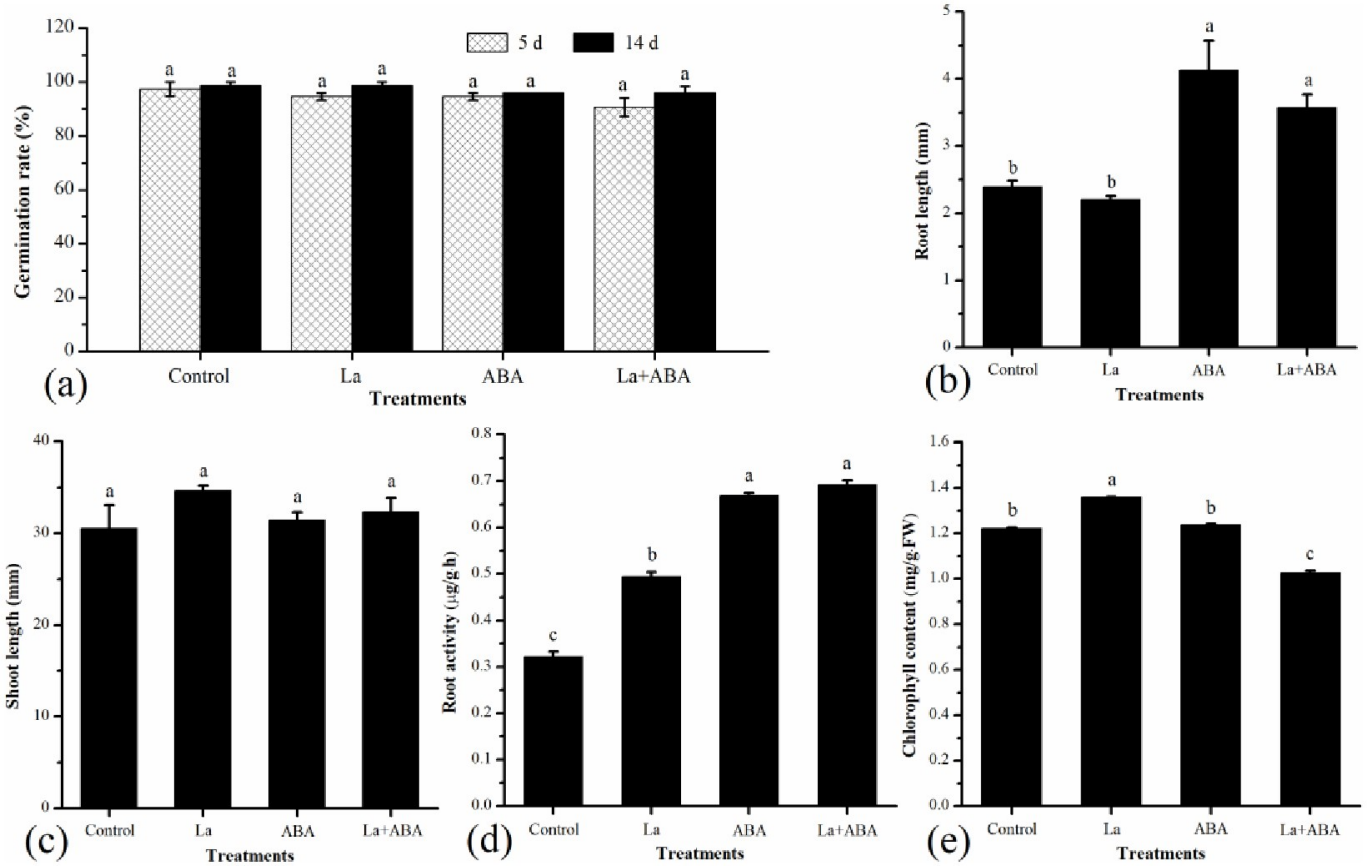
Effects of La and ABA treatments on seed germination and seedling growth in switchgrass. (a) germination rate, (b) root length, (c) shoot length, (d) root activity, (e) chlorophyll content. Note: Means of treatments followed by different letters are significantly different at *P*<0.05.

In order to reveal the effects of La and ABA treatments on seed germination and seedling growth, seed germination rate, root length, shoot length, root activity and chlorophyll content were measured. It was seen that La and ABA had no significant effects on seed germination rate at day 5 and day 14 (Fig 1(a), S1a Table). Following growth for 14 d after germination, the average root length was 2.39 mm in the control experiment, while it was 4.13 mm in the 1/2 MS medium with 10 μM ABA and 3.57 mm in the medium with both 10 μM ABA and 10 μM La(NO_3_)_3_ (Fig 1(b), S1b Table). However, the analysis of shoot length showed that neither La nor ABA had a significant effect on shoot elongation (Fig 1(c), S1c Table)). To further explore the effect of La and ABA on switchgrass root length, and root activity was measured, respectively (Fig 1(d), S1d Table)). The root activity was 0.321 μg/g·h for the control, and it was 50.5% and 107.8% higher than this value in the La and ABA treatments, respectively. The largest rate of increase (115.4%) in root activity was observed in the treatment with La + ABA. The analysis of chlorophyll content of seedling under different treatments showed that the La treatment significantly increased the chlorophyll content (*P*<0.05), which was elevated by 11.5% compared to the control. ABA treatment had no significant difference from the control, while La + ABA treatment lowered the chlorophyll content by 16.4% as compared to the control (Fig 1(e), S1e Table).

### In vitro experiments and quantum chemical calculations

#### The absorbances in different treatments in the in vitro experiments

**Fig 2:**
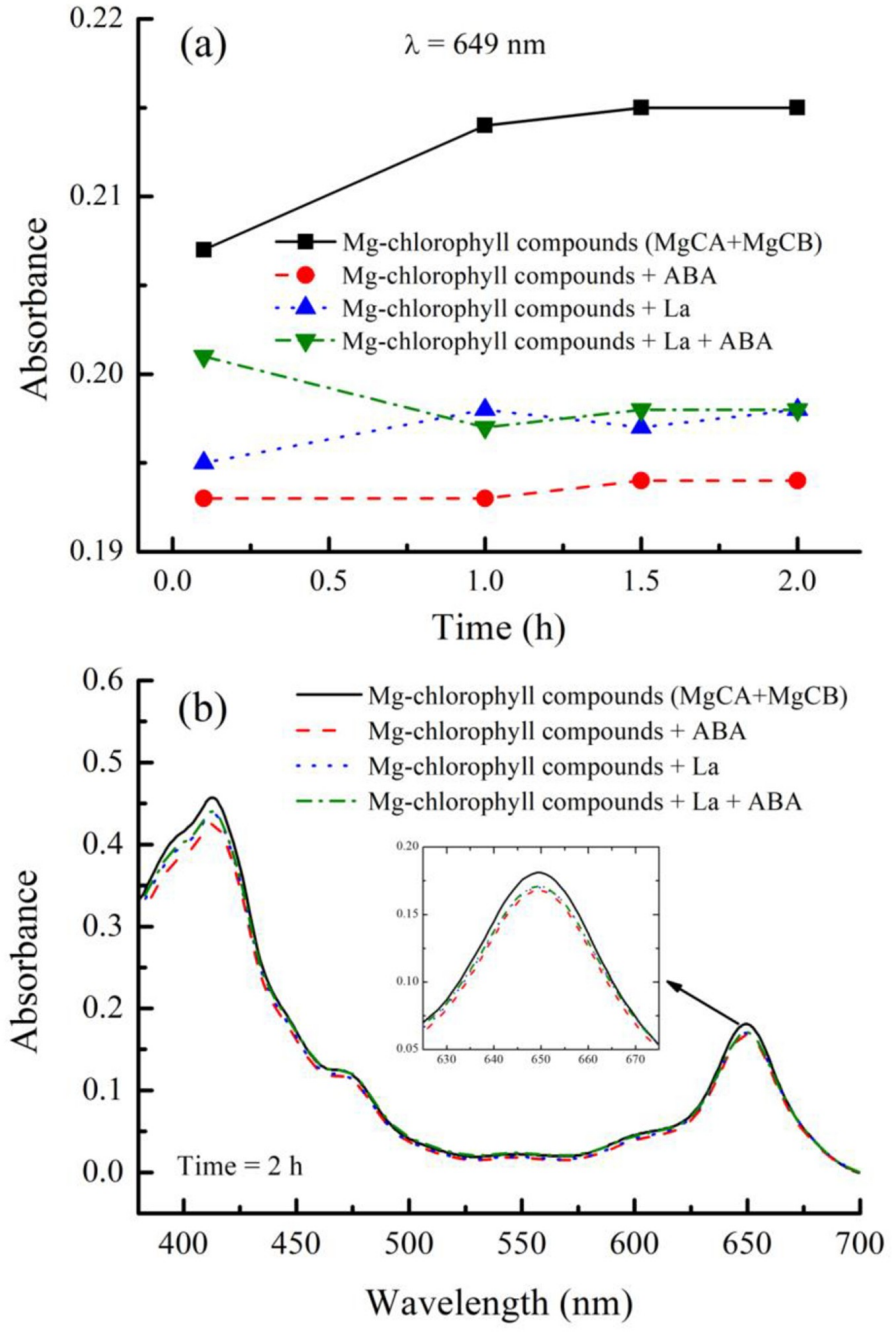
The absorbances in different treatments in the in vitro experiments. (a) The absorbances in the control (MgCA + MgCB), ABA, La, and La + ABA treatments as a function of reaction time, (b) UV-vis spectra in the control (MgCA+MgCB), ABA, La, and La + ABA treatments.

The absorbance and UV-Vis spectra were measured as a function of reaction time, as shown in Fig 2. The results showed that the UV-Vis spectra of the control (Mg-chlorophyll A (MgCA) + Mg-chlorophyll B MgCB), ABA, La, and La + ABA treatments were similar to each other, and all of them had two strong absorption peaks at 413 and 649 nm in the visible region. The absorbance of Mg-chlorophyll compounds was always the greatest, and it increased in the first hour and reached a plateau thereafter. The lowest absorbance was observed in the ABA treatment, remaining nearly unchanged in the first 2 h. The absorbance in the La+ABA treatment was greater than that in the La treatment in the first hour, but the absorbances became nearly the same after the first hour.

#### The optimized structures and Gibbs free energy differences

**Fig 3:**
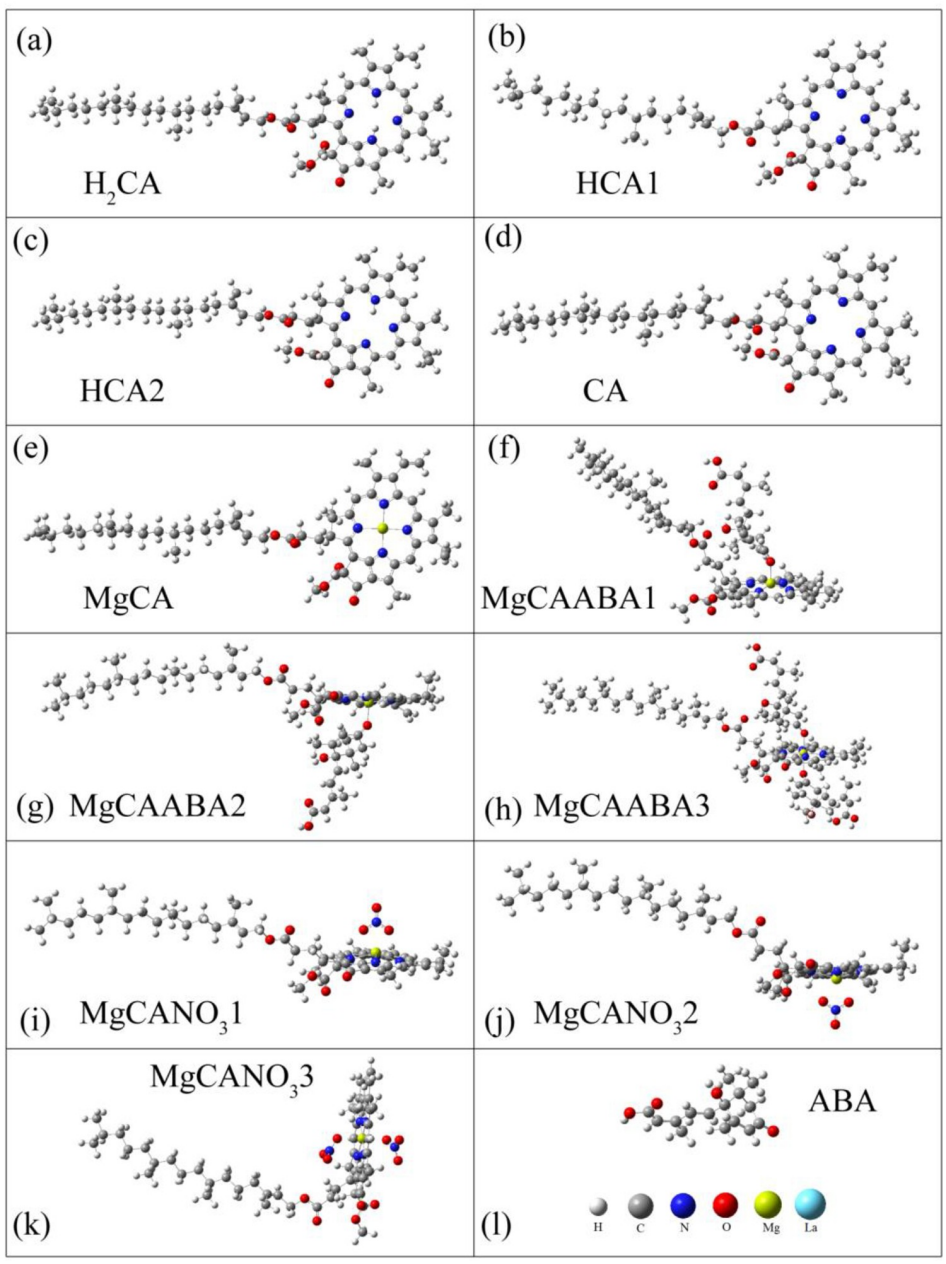
Optimized structures for the reactions between ${\text{NO}}_{3^{-}}$, ABA and Mg-chlorophyll A. (a) Free-base chlorophyll A (H_2_CA), (b-c) monodeprotonated free-base chlorophyll A (HCA1 and HCA2), (d) dideprotonated free-base chlorophyll A (CA), (e) Mg-chlorophyll A (MgCA), (f-h) abscisic acid (ABA) adsorbed onto Mg-chlorophyll A (MgCAABA1, MgCAABA2, and MgCAABA3), (i-k) ${\text{NO}}_{3^{-}}$ adsorbed onto Mg-chlorophyll A (MgCANO_3_1, MgCANO_3_2, and MgCANO_3_3) and (l) abscisic acid (ABA).

**Fig 4:**
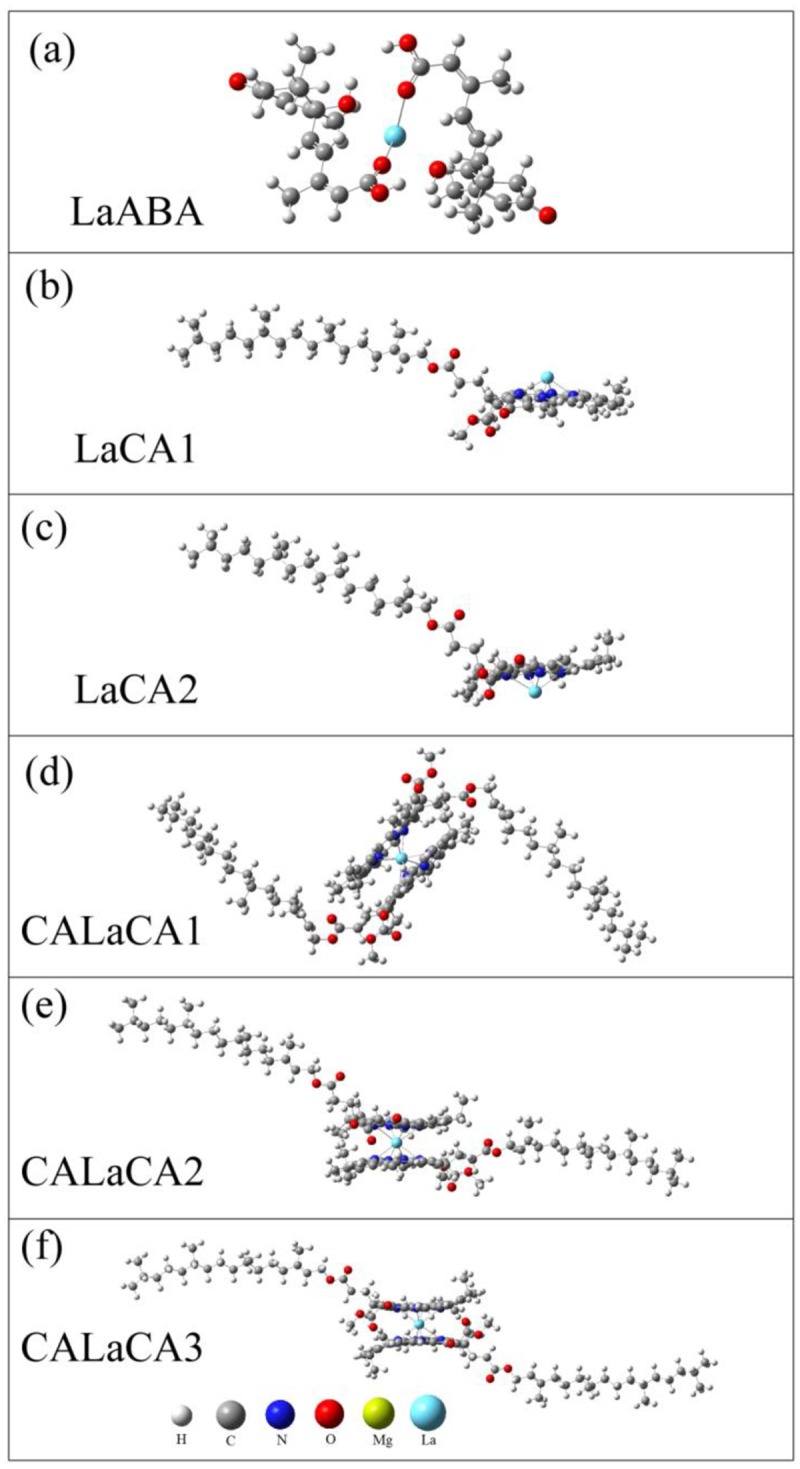
Optimized structures for the reactions between La, ABA and Mg-chlorophyll A. (a) LaABA compound, (b-c) La-monochlorophyll A compounds (LaCA1 and LaCA2), and (d-f) sandwich-type La-bischlorophyll A compounds (CALaCA1, CALaCA2, and CALaCA3).

In the in vitro experiments, the difference of the absorbance should be caused by the reactions between La(NO_3_)_3_, ABA and Mg-chlorophyll compounds. In order to find out the possible products, we systematically investigated the Mg-chlorophyll A (MgCA) + ABA and MgCA + La(NO_3_)_3_ reactions, as well as the reaction of ABA + La(NO_3_)_3_. Considering that part of the MgCA may have decomposed during the reactions, we also optimized the structures of free-base chlorophyll A (H_2_CA), monodeprotonated free-base chlorophyll A (HCA1 and HCA2), and dideprotonated free-base chlorophyll A (CA). It should be mentioned that Mg-chlorophyll B (MgCB), which is another main component of chlorophyll, has a similar structure and property as MgCA. Therefore, MgCB was not investigated in the present work. MgCA consists of a chlorin ring, a central Mg, several attached side chains and a long hydrocarbon tail, as shown in Fig 3. It should be noted that the two sides of the chlorin ring are unequal due to the side chains and the long tail. For the MgCA + ABA reaction, one ABA molecule could be adsorbed onto either side of the chlorin ring, or two ABA molecules could be adsorbed onto both sides of the chlorin ring at the same time. Therefore, three compounds (MgCAABA1, MgCAABA2, and MgCAABA3) could be produced. For the MgCA + La(NO_3_)_3_ reaction, both La and ${\text{NO}}_{3^{-}}$ could react with MgCA. The reaction of MgCA + ${\text{NO}}_{3^{-}}$ was very similar to the MgCA + ABA reaction; therefore, three compounds (MgCANO_3_1, MgCANO_3_2, and MgCANO_3_3) were obtained. For the reaction of MgCA + La, the central Mg could be replaced by La, and two kinds of products were formed, as shown in Fig 4. One kind included the La-monochlorophyll A compounds (LaCA1 and LaCA2), and the other included the sandwich-type La-bischlorophyll A compounds (CALaCA1, CALaCA2, and CALaCA3). In these compounds, La was always located outside of the chlorin ring because the radius of La was larger than that of Mg. Our calculation also showed that one La and two ABA molecules formed a stable La-ABA compound.

**Fig 5:**
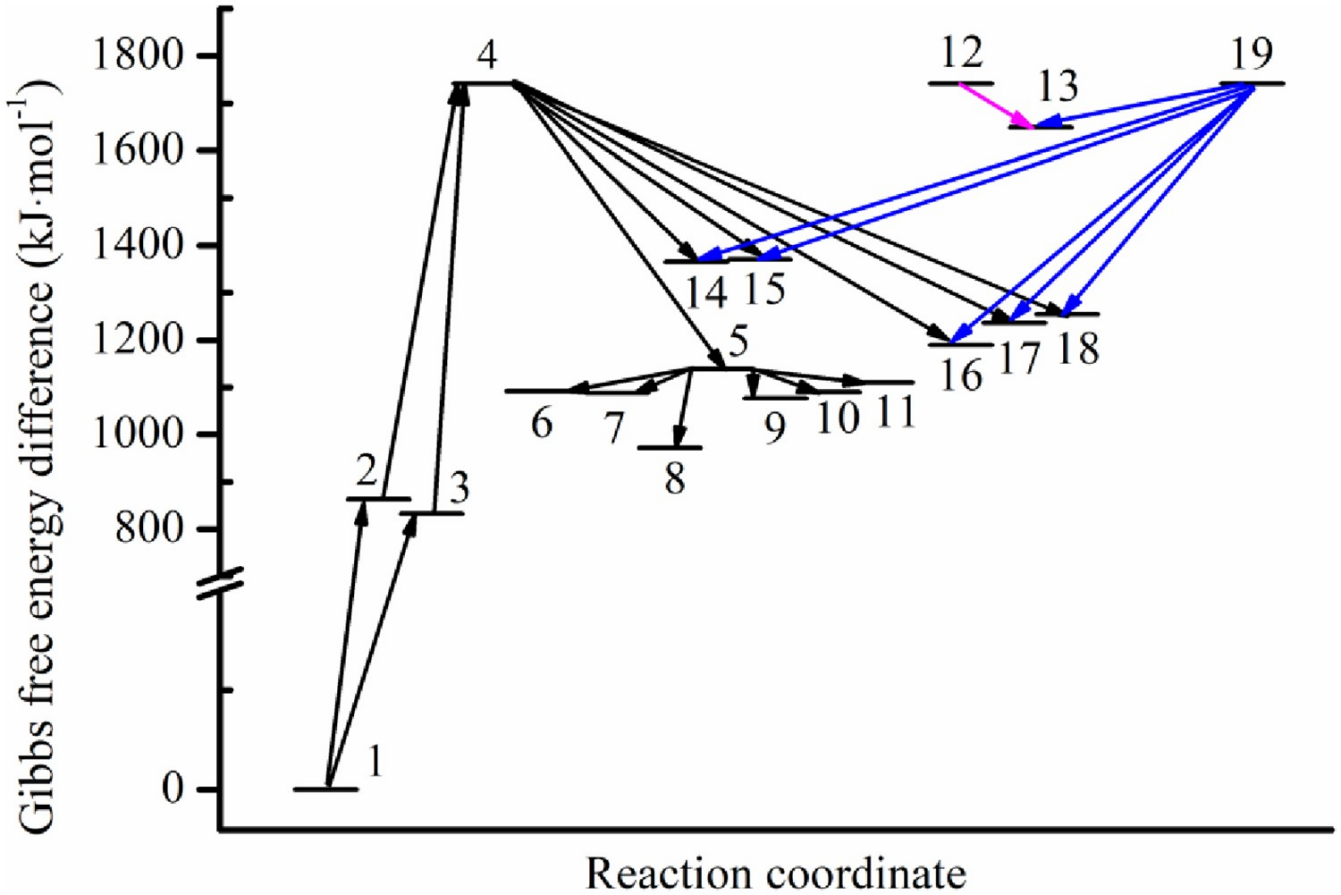
Gibbs free energy differences corresponding to the reactions between different compounds.

For these optimized structures, the Gibbs free energies at 298.15 K and 101 kPa in ethanol were calculated, which could then be used to evaluate whether a given chemical change was thermodynamically possible. The Gibbs free energy differences corresponding to the reactions between different compounds are listed in Table 1 and illustrated in Fig 5.

**Table 1:** Gibbs free energy differences.

Compounds		Gibbs free energy difference (kJ·mol^-1^)
1	H_2_CA	0.00
2	HCA1	863.22
3	HCA2	832.28
4	CA	1741.23
5	MgCA	1138.42
6	MgCAABA1	1091.95
7	MgCAABA2	1087.48
8	MgCAABA3	972.49
9	MgCANO_3_1	1076.19
10	MgCANO_3_2	1089.58
11	MgCANO_3_3	1110.58
12	ABA	1741.23
13	LaABA	1649.07
14	LaCA1	1364.99
15	LaCA2	1370.77
16	CALaCA1	1189.61
17	CALaCA2	1236.61
18	CALaCA3	1254.46
19	La	1741.23

Deprotonation of free-base chlorophyll A (H_2_CA) was a two-step endothermic process. The first step produced monodeprotonated free-base chlorophyll A (HCA1 and HCA2) with an increase in Gibbs free energy of more than 800 kJ·mol^-1^, and the second step formed dideprotonated free-base chlorophyll A (CA) along with another increase of approximately 800 kJ·mol^-1^. The formation of MgCA by Mg and CA was exothermic, and the corresponding decrease in Gibbs free energy was 602.81 kJ·mol^-1^. The adsorption of ABA and ${\text{NO}}_{3^{-}}$ onto MgCA further reduced the Gibbs free energies. The formations of La-monochlorophyll A compounds (LaCA1 and LaCA2) and La-bischlorophyll A compounds (CALaCA1, CALaCA2, and CALaCA3) by La and CA were also exothermic, while the former had higher Gibbs free energies than the latter. In addition, the generation of LaABA compounds by La and ABA decreased the Gibbs free energy by 92.16 kJ · mol^-1^.

#### Simulated electronic absorption spectra

**Fig 6:**
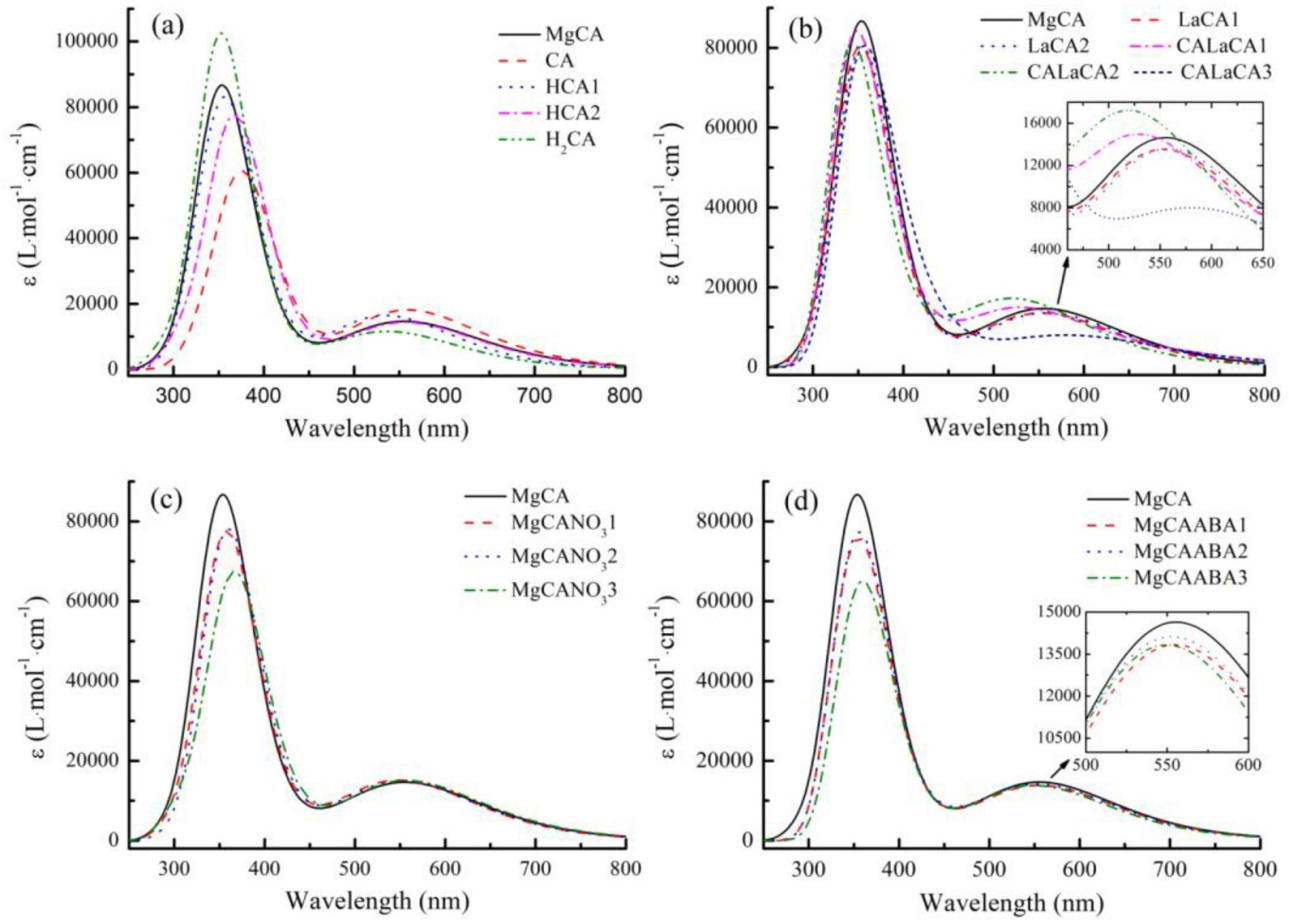
Simulated electronic absorption spectra of different compounds in ethanol. (a) MgCA, CA, HCA1, HCA2, and H_2_CA; (b) MgCA, LaCA1, LaCA2, CALaCA1, CALaCA2, and CALaCA3; (c) MgCA, MgCANO_3_1, MgCANO_3_2, and MgCANO_3_3; (d) MgCA, MgCAABA1, MgCAABA2, and MgCAABA3.

The simulated electronic absorption spectrum of MgCA had two absorption bands (Fig 6). Compared to the experimental results, its short- and long-wavelength absorption peaks were blueshifted by 59 and 93 nm, respectively. These differences were mainly caused by the fact that the MgCA molecule was so large that we could not use the most accurate method to calculate its electronic absorption spectrum, which was very time consuming. Indeed, our electronic absorption spectrum for MgCA was consistent with previous spectra obtained by others [44–46].

Compared to MgCA, dideprotonated free-base chlorophyll A (CA) and monodeprotonated free-base chlorophyll A (HCA1) had a stronger absorption intensity at the long-wavelength absorption band, while free-base chlorophyll A (H_2_CA) possessed a much higher absorption peak at the short-wavelength absorption band.

The electronic absorption spectra of La-monochlorophyll A compounds (LaCA1 and LaCA2) were always weaker than those of MgCA at both absorption peaks. The electronic absorption spectra of La-bischlorophyll A compounds (CALaCA1, CALaCA2 and CALaCA3) were slightly more complicated. For CALaCA3, the absorption intensity decreased at both absorption peaks. For CALaCA1 and CALaCA2, the peak at the short-wavelength absorption band was lower than that of MgCA, but the peak at the long-wavelength absorption band was higher and exhibited a blueshift.

The adsorption of ${\text{NO}}_{3^{-}}$ onto MgCA lowered the absorption peak at the short-wavelength absorption band but barely affected the absorption peak at the long-wavelength absorption band. The interaction of ABA with MgCA reduced the absorption intensity at both the long- and short-wavelength absorption bands.

## Discussion

In the present study, we observed that La and ABA had no significant effect on seed germination, suggesting that the caryopsis covering structure was somewhat restrictive to the movement of macromolecular substances [32], and that ABA could significantly promote the root growth of switchgrass, while La could not alleviate the promotion of root growth by ABA. The root is the major organ for the absorption of water and nutrients. Treatment with an appropriate concentration of lanthanum can improve root activity [47]. The root activity in this paper was reflected by the intensity of root tetrazolium reduction. The results indicated that both the La and ABA treatments enhanced the root activity of switchgrass, but ABA played a more important role.

The chlorophyll content of leaves is an important index that reflects the physiological state of the leaves and corresponds well with photosynthetic capability. Some studies showed that lanthanum could increase the chlorophyll content by inducing the synthesis of the precursor of chlorophyll by promoting the assimilation of certain elements, such as N, P, and Mg [48,49]. Moreover, they speculated that the rare earth metal element was an activator of the enzyme involved in the synthesis of chlorophyll and indirectly improved the synthesis of chlorophyll. Hong *et al*. [33] studied the effect of lanthanum on the chlorophyll of spinach (*Spinacia oleracea*) and found that La improved the assimilation of Mg. Yang *et al*. [50] proved that ABA could improve the chlorophyll content in wheat (*Triticum aestivum*). Our in vivo experiments measured the changes of chlorophyll content under La, ABA and La + ABA treatments. However, the changes of chlorophyll content can be caused by two reasons. One is the change of the total amount of chlorophyll. The other is the change of chlorophyll form. In order to clarify these phenomena, we further implemented in vitro experiments and quantum chemical calculations.

In vivo, La treatment increased the chlorophyll content. However, in vitro, La treatment produced the compounds of La-monochlorophyll A (LaCA1 and LaCA2) and La-bischlorophyll A (CALaCA1, CALaCA2 and CALaCA3), which was consistent with the finding of Mironov [51], and decreased the absorbance of Mg-chlorophyll compounds, which was due to the fact that La-monochlorophyll A and La-bischlorophyll A compounds had lower absorption peaks than Mg-chlorophyll A. This contradiction revealed that La(NO_3_)_3_ should play a role in increasing the total chlorophyll content in vivo because the replacement of Mg with La did not increase the absorbance. Our calculations also showed that La-monochlorophyll A and La-bischlorophyll A compounds had higher Gibbs free energies than MgCA, which indicated that the transformation from MgCA to La-monochlorophyll A or La-bischlorophyll A compounds was thermodynamically unfavorable at 298.15 K and 101 kPa in their standard states. This situation further demonstrated that the increase in chlorophyll content with La treatment in vivo was mainly caused by a stimulatory effect of La rather than the substitution of La for Mg.

ABA treatment had no impact on the chlorophyll content in vivo, but it reduced the absorbance of Mg-chlorophyll compounds in vitro. In vitro, ABA reacted with Mg-chlorophyll compounds, which lowered the absorption peaks. In vivo, ABA also interacted with Mg-chlorophyll compounds, and consequently, the absorbance should have been reduced. Considering that the chlorophyll content remained unchanged with ABA treatment in vivo, more Mg-chlorophyll compounds must have been produced to compensate for the reduction in absorbance caused by ABA. Therefore, ABA should also play a role in promoting the production of Mg-chlorophyll compounds in vivo. Our calculations further showed that the adsorption of ABA onto MgCA decreased the Gibbs free energies (Figs 3, 4 and 5; Table 1), which demonstrated that adsorption was thermodynamically possible.

In vitro, treatment with La + ABA lowered the absorption intensity of Mg-chlorophyll compounds, which was consistent with our findings that both ABA and La(NO_3_)_3_ could react with Mg-chlorophyll compounds and that the products had lower absorption peaks. However, in vivo, treatment with La + ABA reduced the chlorophyll content, which conflicted with our conclusion that both ABA and La(NO_3_)_3_ could stimulate the production of Mg-chlorophyll compounds. In Fig 2(a), we can see that the absorbance in the La + ABA treatment was greater than that in the ABA and La treatments in the first hour. This phenomenon provided evidence that La should react with ABA and may form LaABA compound (Figs 3, 4 and 5). One hour later, the absorbance in the La + ABA treatment became nearly the same as that in the La treatment, which demonstrated that some of the LaABA compound were converted into La-monochlorophyll and La-bischlorophyll compounds. Our calculations predicted that the Gibbs free energy would decrease from La to LaABA compound, and it also fell from LaABA compound to La-monochlorophyll and La-bischlorophyll compounds; thus, both the formation of LaABA compound and the conversion were thermodynamically possible. In the in vivo experiment, adding ABA and La(NO_3_)_3_ to culture medium produced LaABA compound. When the LaABA compound remained in the culture medium, the concentrations of ABA and La(NO_3_)_3_ were reduced in vivo, which weakened the stimulatory effects of ABA and La(NO_3_)_3_. However, although ABA and La(NO_3_)_3_ were at low concentrations in vivo, they still reacted with Mg-chlorophyll compounds, which lowered the absorbance. The overall effect was that the formation of LaABA compound allowed the stimulatory effect to be overcome by the inhibitory effect, in turn reducing the absorbance. As a result, the measured chlorophyll content decreased in vivo.

Overall, our in vivo and in vitro experiments, together with the quantum chemical calculations, demonstrated that both La and ABA could promote the production of chlorophyll in vivo, although they also weakened the absorption intensity of Mg-chlorophyll compounds by reacting with them. When La and ABA were used at the same time, the competition between the effects of promoting chlorophyll production and reducing chlorophyll absorbance became complicated because the ABA and La formed LaABA compound, which markedly reduced the concentrations of ABA and La.

The plant hormone ABA has many important functions in plant growth and development, especially in plant stress responses. As both La and ABA have positive effects on plant stress responses [52,53], it would be interesting to further study the mechanisms of La and ABA interactions in switchgrass stress responses at the molecular level.

## Conclusions

This work demonstrates that La and ABA can interact with each other to affect seedling growth of switchgrass. Both La and ABA enhance the root activity of switchgrass, but ABA is more efficient. ABA also accelerates the root elongation. Moreover, La significantly increases the chlorophyll content, whereas La + ABA decreases the chlorophyll content as compared to the control. The underlying mechanism is inferred from in vivo experiments, in vitro experiments and quantum chemical calculations. The results indicate that both ABA and La can stimulate the production of chlorophyll, while they also could react with chlorophyll to produce La-monochlorophyll, La-bischlorophyll, and ABA adsorbed chlorophyll, which have lower absorbance. ABA + La treatment forms LaABA compound, which markedly reduces the concentrations of ABA and La and further influences the competition between promoting chlorophyll production and reducing the absorbance of chlorophyll.

## Supporting information

S1 Table (DOCX)
