# Supplementary material for: Lanthanum and abscisic acid coregulate chlorophyll production of seedling in switchgrass
Source: PLoS One. 2020 May 5;15(5):e0232750. doi: 10.1371/journal.pone.0232750 (PMC7199945; doi:10.1371/journal.pone.0232750)
Supplement: S1 Table — (DOCX) [file pone.0232750.s001.docx]

Supporting Information

**Lanthanum and abscisic acid coregulate chlorophyll production of seedling in switchgrass**

Xueqing He*, Pei You, Yunfu Sun

College of Grassland Agriculture, Northwest A&F University, Yangling, Shaanxi Province, China

* Corresponding author

E-mail: [hexueqing@nwsuaf.edu.cn](mailto:hexueqing@nwsuaf.edu.cn) (XH)

The data of seed germination rate, root length, shoot length, root activity and chlorophyll content by different treatments is listed in S1 Table.

**S1a Table. Effect of La and ABA treatments on seed germination in switchgrass**

| Treatments | Germination rate (%) | |
| --- | --- | --- |
|  | 5 d | 14 d |
| Control-1 | 100 | 100 |
| Control-2 | 100 | 100 |
| Control-3 | 92 | 96 |
| La-1 | 92 | 96 |
| La-2 | 96 | 100 |
| La-3 | 96 | 100 |
| ABA-1 | 96 | 96 |
| ABA-2 | 96 | 96 |
| ABA-3 | 92 | 96 |
| La+ABA-1 | 92 | 100 |
| La+ABA-2 | 96 | 96 |
| La+ABA-3 | 84 | 92 |

**S1b Table. Effect of La and ABA treatments on root length in switchgrass**

| Treatments | Root length (mm) | | | | | | | | | | |
| --- | --- | --- | --- | --- | --- | --- | --- | --- | --- | --- | --- |
|  | 1 | 2 | 3 | 4 | 5 | 6 | 7 | 8 | 9 | 10 | Average |
| Control-1 | 5.1 | 2.2 | 3.3 | 1.9 | 1.2 | 2.3 | 2.1 | 2.2 | 0.9 | 1.1 | 2.23 |
| Control-2 | 1.2 | 2.8 | 3.2 | 1.9 | 2.6 | 2.1 | 4.3 | 1.9 | 1.5 | 4.1 | 2.56 |
| Control-3 | 3.1 | 4.2 | 1.1 | 1.2 | 1.3 | 4.4 | 1.3 | 1.0 | 0.2 | 5.1 | 2.39 |
| La-1 | 2.1 | 1.6 | 1.9 | 1.5 | 1.8 | 1.7 | 2.2 | 2.3 | 4.7 | 1.1 | 2.09 |
| La-2 | 2.4 | 2.7 | 3.6 | 2.8 | 1.1 | 1.0 | 2.2 | 2.2 | 3.1 | 1.7 | 2.28 |
| La-3 | 2.0 | 2.7 | 3.6 | 2.8 | 1.1 | 1.0 | 2.2 | 2.2 | 3.1 | 1.7 | 2.24 |
| ABA-1 | 7.7 | 6.9 | 7.2 | 2.7 | 6.0 | 4.2 | 1.6 | 4.5 | 6.1 | 3.0 | 4.99 |
| ABA-2 | 5.1 | 4.3 | 2.1 | 6.1 | 3.4 | 2.2 | 3.3 | 1.9 | 5.3 | 1.8 | 3.55 |
| ABA-3 | 7.2 | 5.1 | 1.1 | 2.1 | 3.3 | 7.5 | 2.4 | 2.5 | 4.2 | 3.1 | 3.85 |
| La+ABA-1 | 5.1 | 1.1 | 1.9 | 4.0 | 6.2 | 4.1 | 3.2 | 1.9 | 2.2 | 3.1 | 3.28 |
| La+ABA-2 | 8.2 | 2.2 | 4.3 | 4.5 | 2.9 | 5.2 | 1.3 | 1.9 | 3.6 | 5.2 | 3.93 |
| La+ABA-3 | 4.6 | 5.1 | 2.5 | 1.6 | 2.5 | 5.6 | 5.1 | 4.8 | 2.0 | 1.3 | 3.51 |

**S1c Table. Effect of La and ABA treatments on shoot length in switchgrass**

| Treatments | Shoot length (mm) | | | | | | | | | | |
| --- | --- | --- | --- | --- | --- | --- | --- | --- | --- | --- | --- |
|  | 1 | 2 | 3 | 4 | 5 | 6 | 7 | 8 | 9 | 10 | Average |
| Control-1 | 31.1 | 32.1 | 33.1 | 34.1 | 36.1 | 40.1 | 35.1 | 38.1 | 39.1 | 37.1 | 35.6 |
| Control-2 | 22.9 | 32.1 | 19.2 | 33.2 | 28.0 | 29.7 | 26.4 | 22.8 | 30.9 | 31.8 | 27.7 |
| Control-3 | 21.8 | 39.2 | 29.8 | 34.1 | 22.0 | 22.1 | 32.8 | 21.9 | 25.5 | 34.2 | 28.34 |
| La-1 | 38.7 | 28.9 | 33.9 | 34.3 | 33.0 | 42.1 | 28.8 | 38.9 | 36.3 | 35.8 | 35.7 |
| La-2 | 33.2 | 34.1 | 40.0 | 33.1 | 35.8 | 35.9 | 35.2 | 37.1 | 34.2 | 23.0 | 34.16 |
| La-3 | 33.2 | 34.1 | 40.0 | 33.1 | 35.8 | 35.9 | 35.2 | 37.1 | 34.2 | 23.0 | 34.16 |
| ABA-1 | 34.6 | 27.4 | 40.7 | 32.1 | 39.2 | 28.0 | 30.3 | 28.9 | 27.9 | 34.9 | 32.4 |
| ABA-2 | 30.9 | 27.8 | 28.9 | 31.8 | 31.0 | 33.2 | 40.0 | 34.9 | 37.2 | 26.0 | 32.17 |
| ABA-3 | 34.0 | 33.7 | 13.1 | 30.0 | 23.8 | 30.4 | 32.0 | 35.1 | 31.7 | 33.4 | 29.72 |
| La+ABA-1 | 29.9 | 26.3 | 37.1 | 31.6 | 29.8 | 22.5 | 23.6 | 32.1 | 26.4 | 34.3 | 29.36 |
| La+ABA-2 | 34.9 | 28.3 | 36.9 | 38.8 | 44.8 | 29.2 | 29.1 | 32.7 | 31.5 | 35.9 | 34.21 |
| La+ABA-3 | 30.0 | 34.2 | 31.9 | 34.7 | 33.1 | 34.5 | 38.9 | 34.1 | 29.9 | 34.0 | 33.53 |

**S1d Table. Effect of La and ABA treatments on root activity in switchgrass**

| Treatments | Absorbance  (485 nm) | Tetrazolium reduction  (μg) | Reduction strength of tetrazolium (μg·g^-1^·h^-1^ ) |
| --- | --- | --- | --- |
| Control-1 | 0.071 | 0.543 | 0.302 |
| Control-2 | 0.076 | 0.585 | 0.325 |
| Control-3 | 0.079 | 0.610 | 0.339 |
| La-1 | 0.109 | 0.862 | 0.479 |
| La-2 | 0.116 | 0.921 | 0.512 |
| La-3 | 0.112 | 0.887 | 0.493 |
| ABA-1 | 0.148 | 1.190 | 0.661 |
| ABA-2 | 0.152 | 1.223 | 0.680 |
| ABA-3 | 0.149 | 1.198 | 0.666 |
| La+ABA-1 | 0.151 | 1.215 | 0.675 |
| La+ABA-2 | 0.158 | 1.274 | 0.708 |
| La+ABA-3 | 0.155 | 1.249 | 0.694 |

**S1e Table. Effect of La and ABA treatments on chlorophyll content of seedling in switchgrass**

| Treatments | Absorbance | | Chl a  (mg/L) | Chl b  (mg/L) | Total Chl  (mg/L) | Chlorophyll content(mg/g) |
| --- | --- | --- | --- | --- | --- | --- |
|  | 665 nm | 649 nm |  |  |  |  |
| Control-1 | 0.343 | 0.071 | 3.904 | 0.954 | 4.858 | 1.214 |
| Control-2 | 0.346 | 0.076 | 3.934 | 0.982 | 4.916 | 1.229 |
| Control-3 | 0.345 | 0.079 | 3.926 | 0.964 | 4.890 | 1.223 |
| La-1 | 0.379 | 0.109 | 4.294 | 1.144 | 5.438 | 1.360 |
| La-2 | 0.379 | 0.116 | 4.294 | 1.144 | 5.438 | 1.360 |
| La-3 | 0.380 | 0.112 | 4.307 | 1.137 | 5.444 | 1.361 |
| ABA-1 | 0.334 | 0.148 | 3.741 | 1.203 | 4.943 | 1.236 |
| ABA-2 | 0.335 | 0.152 | 3.760 | 1.169 | 4.929 | 1.232 |
| ABA-3 | 0.337 | 0.149 | 3.776 | 1.206 | 4.982 | 1.245 |
| La+ABA-1 | 0.292 | 0.151 | 3.350 | 0.696 | 4.046 | 1.011 |
| La+ABA-2 | 0.294 | 0.158 | 3.365 | 0.733 | 4.098 | 1.025 |
| La+ABA-3 | 0.297 | 0.155 | 3.389 | 0.797 | 4.176 | 1.044 |
